# Author's Reply

**DOI:** 10.1371/journal.pmed.0020191

**Published:** 2005-06-28

**Authors:** Deborah Hayden

**Affiliations:** San Anselmo, CaliforniaUnited States of America

The excellent article by Jordan Paradise, Lori B. Andrews, and colleagues, “Ethics. Constructing Ethical Guidelines for Biohistory” [1], neither advocates nor argues against biohistorical research; instead, it points out that such investigations are currently taking place without guidelines—ethical, scientific, moral, or religious. The question remains: if such guidelines were to be established, what individuals, institutions, governments, medical examiners, family members, or intrepid biographers are to be given permission? Who is to decide what is “historically significant”? Not to mention the meta-question: who is to decide who is to decide? I apologize to the authors if my brief comments [2] implied that they took a position on this issue.
